# Polysubstance use among patients receiving opioid agonist maintenance treatment in Belarus: source-dependent underreporting and implications for care

**DOI:** 10.1192/j.eurpsy.2026.10484

**Published:** 2026-07-31

**Authors:** U. Pikirenia, Y. Volkau

**Affiliations:** 1 Substance Use Disorders Department, Psychiatric Hospital in Frombork, Frombork, Poland; 2 Low threshold services, NGO “Positive Movement”, Minsk, Belarus

## Abstract

**Introduction:**

Opioid agonist maintenance treatment (OAMT) reduces mortality and HIV-related harms, yet concurrent substance use remains common and undermines outcomes. Robust estimates are essential for service planning and overdose prevention.

**Objectives:**

To quantify recent substance use among OAMT patients in Belarus, compare prevalence by data-collection route, and examine associations with treatment characteristics.

**Methods:**

Cross-sectional survey (February – March 2021) of OAMT patients using an anonymised questionnaire. Substances queried: nicotine, alcohol (light/strong), cannabinoids, synthetic cathinones, amphetamines, off-label sedatives/hypnotics (benzodiazepines, Z-drugs), anticholinergics (e.g., diphenhydramine), opioids, and synthetic cannabinoids. Recency of last use was coded ordinally (day→>12 months/never). Data were collected via three routes: online, peer consultants, or clinic staff. Descriptives, χ² tests (with ε²), Spearman correlations, and t-tests were used (α=0.05). Only anonymised records were analysed.

**Results:**

N=199 (mean age 41.3 years; ~57% male). In the previous year, 87.9% used nicotine (85.9% daily), 62.2% light alcohol, 46.9% sedatives/hypnotics, 44.1% strong alcohol, 21.6% cannabis, 19.3% anticholinergics, 18.1% synthetic cathinones, 9.9% opioids, 8.0% amphetamines, and 0.5% synthetic cannabinoids.

The mean number of substance groups used in 12 months differed by collection route: online 3.8, peer 3.5, staff 1.8 (χ² = 44.53, df = 2, p < 0.0001, ε² = 0.24); for illegal substances: 1.6, 1.5, 0.4 (χ² = 40.72, df = 2, p<0.0001, ε² = 0.22).

Longer OAMT duration correlated with fewer substance groups in the last year (ρ=−0.203, p=0.007) and fewer illegal substances (ρ=−0.242, p=0.001). No differences by methadone vs buprenorphine or by prescribed dose.

Gender differences were found for cannabis (men 30.6% vs women 9.1%) and opioids (men 19.1% vs women 2.1%; p=0.006).

**Conclusions:**

Polysubstance use, especially nicotine, alcohol, sedatives/hypnotics, and synthetic cathinones, is prevalent among Belarusian OAMT patients, but prevalence is substantially under-reported when disclosures are made to clinic staff. Findings support non-punitive, confidential assessment pathways (online/peer-mediated), routine screening for sedatives and alcohol, targeted harm-reduction/overdose prevention, and tobacco harm-reduction support. Sustained engagement with OAMT appears protective against broader substance involvement; services should prioritise retention while addressing polysubstance risks.

**Disclosure of Interest:**

None Declared

